# Janus Nematic Colloids with Designable Valance

**DOI:** 10.3390/ma7064272

**Published:** 2014-05-30

**Authors:** Simon Čopar, Miha Ravnik, Slobodan Žumer

**Affiliations:** 1 Faculty of Mathematics and Physics, University of Ljubljana, Jadranska 19, Ljubljana 1000, Slovenia; E-Mails: simon.copar@fmf.uni-lj.si (S.Č.); miha.ravnik@fmf.uni-lj.si (M.R.); 2 Jozef Stefan Institute, Jamova 34, Ljubljana 1000, Slovenia

**Keywords:** Janus colloids, nematic liquid crystal, surface anchoring, valence

## Abstract

Generalized Janus nematic colloids based on various morphologies of particle surface patches imposing homeotropic and planar surface anchoring are demonstrated. By using mesoscopic numerical modeling, multiple types of Janus particles are explored, demonstrating a variety of novel complex colloidal structures. We also show binding of Janus particles to a fixed Janus post in the nematic cell, which acts as a seed and a micro-anchor for the colloidal structure. Janus colloidal structures reveal diverse topological defect configurations, which are effectively combinations of surface boojum and bulk defects. Topological analysis is applied to defects, importantly showing that topological charge is not a well determined topological invariant in such patchy nematic Janus colloids. Finally, this work demonstrates colloidal structures with designable valence, which could allow for targeted and valence-conditioned self-assembly at micro- and nano-scale.

## 1. Introduction

Colloidal dispersions with particles of pre-designed shape, surface, size distribution, or bulk material properties are today one of the prospective routes towards new micro- and nano-functional materials [1,2]. Janus colloids are the type of colloids, where particles have specially designed surfaces, characterized by distinct surface patches of diverse modalities. The classical example of Janus colloids, as proposed by de Gennes [3], are colloidal particles with different chemical makeups on their two hemispheres, for example, one side of the particle being hydrophilic whereas the other hydrophobic. Even such relatively simple Janus colloids exhibit a variety of ordered and disordered structures [4,5]. More generally, Janus particles can be polymeric, inorganic, and polymeric–inorganic, and each kind of Janus particles can be spherical, dumbbell-like, half raspberry-like, cylindrical, disk-like, or any of a variety of other shapes [6,7]. Offering such diverse versatility in the surface functionality, morphology, shape, and chemical composition, the Janus particles indicate a range of possible applications, including as solid surfactants, optical probes for microrheology, self-assembled micro-materials, magnetic and magnetic–fluorescent bifunctional materials, and optical and catalytic materials [6,8]. 

A new and in many ways unique functionalisation of Janus colloids is achieved by introducing internal orientation—liquid crystalline—order into the Janus dispersions. This can be achieved by either (i) designing macromolecules or particles with internal Janus liquid crystalline order [9,10] or (ii) by dispersing Janus particles into a liquid crystal [11,12,13]. Under the first approach, the manipulation of the structural fragments of the macromolecules or the topological defects allows for variation of physical properties and thus potential applications, including possibly in the direction of functional complexity found in living systems. Under the second approach, solid particles with one hemisphere homeotropic (perpendicular) and one hemisphere planar surface anchoring are dispersed in nematic liquid crystals, inducing distortions in the nematic field of Janus-type [11]. These distortions were shown to cause attraction of particle pairs into pairs, with the potential strongly dependent on the relative alignment of the hemispheres. The relative strengths of the anchoring on the two hemispheres was shown to change the stable *orientation* of the Janus particles relative to the far-field nematic director, by affecting the bistability of the two possible orientations of the half-planar-half-homeotropic particles. And finally, by using laser illumination, the Janus particles in nematic were demonstrated to act as rotators when exposed to the laser filed, with the frequency increasing with the increasing intensity of light [12]. Recently, also defect topologies in a nematic liquid crystal near a patchy colloid were explored using isothermal-isobaric Monte Carlo simulations [13].

In this paper, we go beyond the classical two-hemisphere nematic Janus colloids, and demonstrate generalized nematic Janus colloids with multiple and diverse configurations of surface patches with different—*i.e.*, homeotropic and planar—surface anchoring. By using mesoscopic numerical modeling, we explore multiple morphologies of surface patches—*i.e.*, types of Janus particle—and demonstrate a variety of novel complex colloidal structures, based exactly on the multi-valent properties of these designed Janus particles. We show also a combined system of Janus particles and fixed Janus posts (pillars), which act as seeds and micro-anchors for the structures of diverse complexity. The nematic Janus colloidal structures reveal diverse topological defect structures which effectively, prove to be combinations of surface boojum defects and bulk −1/2 disclination lines. Topological analysis is applied to such defects, importantly showing that topological charge is not a well determined topological invariant in such patchy nematic Janus colloids, acquiring various values for same particle type. 

## 2. Results and Discussion

**Figure 1:**
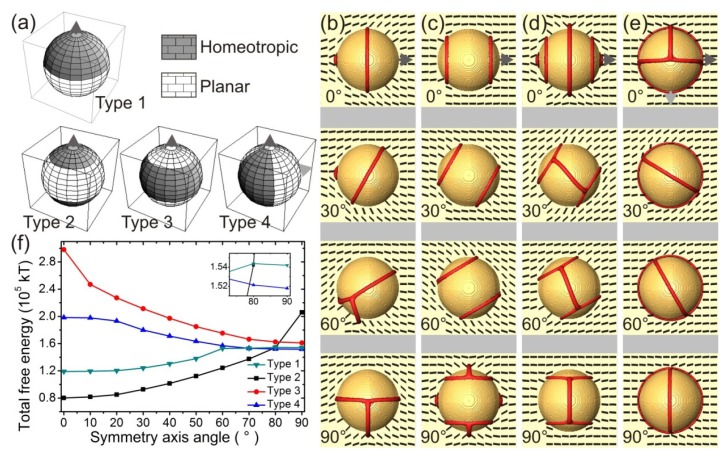
Janus particle in nematic liquid crystal with diverse morphologies of surface anchoring. (**a**) Janus Particles with various configurations of surface patches (Type 1–4) of homeotropic and degenerate planar surface anchoring; (**b**–**e**) Type 1–4 particles in homogeneously aligned planar nematic cell at various angles with respect to the far-field nematic direction. The red isosurfaces denote regions of decreased nematic order, *i.e.*, boojums, seams between patches, and defect arcs through the bulk; (**f**) Total free energy of various-type Janus particles for different particle orientations shown in (**b**–**e**). Particles of types 1 and 2 align with the symmetry axis parallel to the far-field.

The simplest design of a particle with heterogeneous anchoring separates the particle’s surface into two hemispheres with different anchoring conditions, *i.e.*, one homeotropic and one with planar degenerate surface anchoring (see Figure 1a, Type 1). Typically, this type of particle surface morphology is easiest to manufacture and was already reported in liquid crystals [11,12,13]. To extend this idea further, and really explore the role of surface morphology, we increased the number of distinct patches on the particle surface and used numerical simulations to probe the orientation dependence of particle’s interaction with homogeneous nematic field (for more on modeling and theory, see Section 3). In nematics, the elastic interaction is complemented and sometimes superseded by topological interactions, mediated by topological defects—lines and points of undetermined molecular orientation. This makes the interaction not only anisotropic, but creates hysteresis and multiple metastable states. The capability of particles forming “covalent” bonds via entangled defects, explored before for not-Janus homeotropic-anchoring-only particles with great success [14,15,16], can be exploited for stabilization of designed particle aggregates.

We used two surface types: homeotropic and planar degenerate in our construction of higher-valence particles (Figure 1a). The standard two-hemisphere capped Janus particle in different orientation to the far-field is shown in (Figure 1b). Snapshots of the director profile (in black) and corresponding defects (in red) are presented for angles 0°, 30°, 60°, and 90° of the symmetry axis with respect to the far-field director. The particle induces a single boojum when aligned along the symmetry axis, but when rotated beyond 60°, obtains a −1/2 defect line arc, tethered to the surface at the line separating the two Janus hemispheres. The free energy varies with rotating the particle, inducing a torque that restores the particle to the equilibrium state. Interestingly, two rotational minima are observed: the global stable minimum at 0° and a metastable minimum at 90° giving bistability of the two possible particle orientations. By changing the planar and homeotropic anchoring strengths and their relative ratio one can control the relative stability or metastability of the two minima.

Figure 1c–e shows results for generalized Janus particles with more complex morphologies of the surface patches. Type 2 particles have caps with homeotropic surface anchoring and a waist with degenerate planar anchoring, Type 3 has planar caps and a homeotropic waist, and Type 4 have planar and homeotropic stripes alternating in meridional wedges. When rotating the particles, the defect configurations vary by typically either opening the defect cores or generating −1/2 defect arcs. From the total free energy rotation profiles (Figure 1f) one can see that all three configurations have their free energies in a roughly similar range, but with different equilibrium orientations. The equilibrium particle orientations are sensitive to relative strengths of the homeotropic and planar anchoring which could be used for optimization of desired particle orientations. To generalize the behavior of colloidal particles with patterned surfaces, one can consider them as superpositions of homeotropic-only and planar-only anchoring particles. Bulk pieces of the director field bordering to proper surface regions of the “single-anchoring particle” are to be glued together to tile in the surface of a patterned particle in order to build the director profile around a given patterned particle.

**Figure 2:**
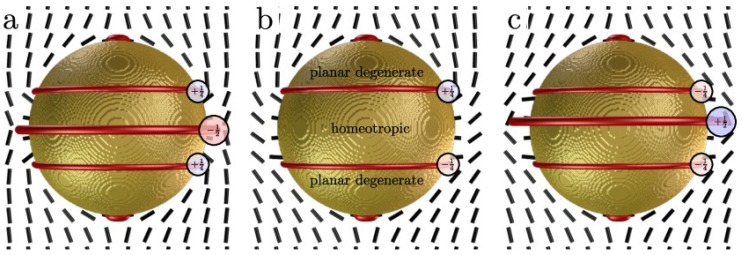
Ambiguity of topological charge in Janus nematic colloids. A simple composite particle with planar degenerate poles and homeotropic equator can select from three director configurations at the seams, causing the resulting defect charge (rank, [19]) to be either (**a**) +1; (**b**) 0; or (**c**) −1. The ±1 structures require a defect ring defect which is energetically unfavorable and makes these structures metastable or unstable relative to the 0 charge structure. The seams between the patches can be considered as surface defect lines with a quarter winding number.

These generalized Janus nematic colloids show a variety of complex topological defect structures in the nematic, which are typically generalized by considering a topological invariant called topological charge [17,18], or the geometry-aware generalization called the defect rank [19]. The conservation of the topological charge ascribed to the particle generally—in non-Janus nematic colloids—governs the creation of all defects accompanying the particle, such as point defects, Saturn rings and boojums. However, around Janus particles the line defects do not generally form closed loops, as seen in Figure 1 where defect arcs are observed. At the seams between patches of different surface anchoring, the elastic free energy density is typically increased, turning the seams into effective surface defects. Moreover, these defect seams naturally attract point defects, boojums and act as a natural tethering spots for the defect lines or loops which then form defect arcs. As a consequence, the particle itself no longer has a fixed topological charge and in the case of tethering, no topological charge can be assigned at all because the director field has singularities at the tethering points. Such particles have degrees of freedom available to minimize the elastic stress surrounding it, as they are not bound by strict topological constraints. The freedom comes from the choice of interpolation of the director field at the seams. The ambiguity of topological charge in Janus nematic colloids is demonstrated in Figure 2. For instance, the particle with planar caps at the poles, can assume three states with different topological charges (Figure 2). The seams can be thought to carry a quarter winding number in two-dimensional the cross section, as the director makes a quarter of a full turn there. However, this turn can be either clockwise or counter-clockwise (−1/4 or +1/4) and the nematic director surrounding the particle as a whole can be in either of the two configurations. The particle can be in a state with topological charge +1 with surrounding Saturn ring (Figure 2a), a charge-neutral state (Figure 2b) or a state with topological charge −1 with an anti-Saturn ring (Figure 2c). Generally, the charge neutral state will expectedly have lowest free energy, because it minimizes elastic stress and the cost of the defect core, and is therefore most likely to be observed in experiments. The states with +1 and −1 topological charge are either metastable or entirely unstable. If the particle is allowed to rotate, states with defect arcs and ill-defined topological charge are also observed (as in Figure 1d). This mechanism is universal and the more patches a particle has, the less elastic deformation it imparts to its surroundings. In the limit of many patches, the particle can be considered having a “rough” surface with effectively no anchoring. The only thing that remains is the reduced degree of order at the surface, which could give rise to wetting and depletion forces.

**Figure 3:**
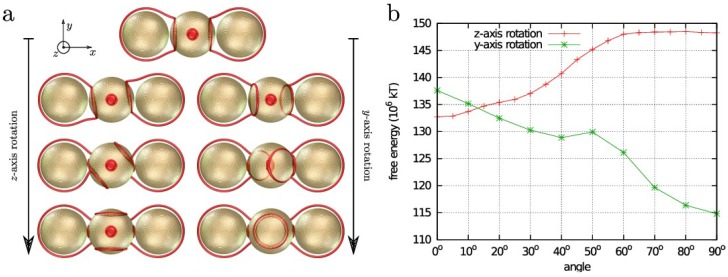
Designing colloidal superstructures with Janus particles. The bivalent Janus particle binds two non-Janus homeotropic particles by tethering their Saturn ring defects to the seams around the caps. The Janus particle in the desired orientation with the symmetry axis in the *x* direction is stable under rotations around the vertical (*z*) axis, but unstable under rotations about the horizontal axis (*y*). Its lowest energy state aligns the caps with the external director along the *z* axis (**b**). The stable configuration of the superstructure is shown in (**a**, bottom right).

Morphology of the surface patches could be related to the “valence” of the particle for forming structures, by imparting a desired symmetry in the arrangement of the patches. Figure 3 demonstrates the stability of a cluster containing a Janus “core” with two homeotropic caps and a planar waist and two homeotropic-only “satellite” particles. The seams of the Janus particle provide tethering points that bind the Saturn rings of the satellite particles to the core if the caps of the Janus particle are facing the satellites (Figure 3b). The particles are confined to a thin homeotropic cell, which prevents them from arranging into a chain along the direction of the field, which is a preferred arrangement in the bulk. However, the orientation with caps toward the satellite particles is unstable. The caps tend to orient along the uniform bulk director set by the homeotropic cell boundaries (see Figure 3). In our broader testing of different Janus configurations, we discovered this instability is universal, because the homeotropic “pull” of the uniform ambient field overpowers the binding to the satellite particles, except in the configuration of the chain along the uniform field, when these two interactions are parallel.

**Figure 4:**
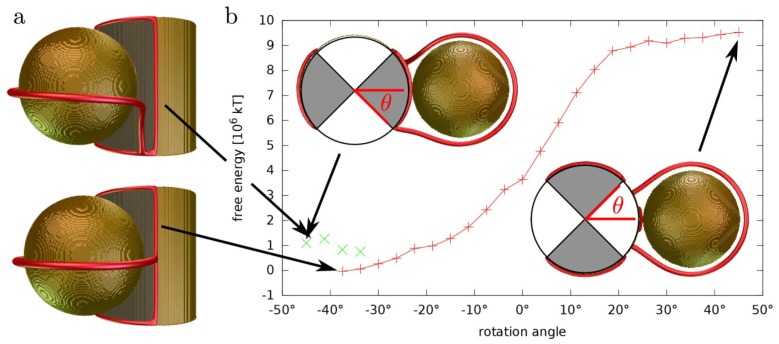
A homeotropic non-Janus particle bound to a Janus post within a nematic cell. The Janus post is segmented into 4 alternating patches of homeotropic (shaded) and planar degenerate anchoring. (**a**) We observe two bound states (one stable, one metastable) when the particle is centred next to the homeotropic patch. The metastable state (upper panel) has the defect line tethered to the bottom corner of the post, whereas in the stable state the defect line tethers to the Janus seams of the post; (**b**) Free energy study of the particle binding shows that the maximum or minimum of the free energy are found when the particle is positioned exactly symmetrically at the homeotropic or planar patch, respectively.

The orientation instability becomes irrelevant if a Janus particle is replaced with a fixed vertical Janus post which then serves as a micro-anchor for the colloidal structure. Indeed, recent experiments with microfabricated arrays of microposts provide a promising starting ground for physical realization of such Janus posts [20]. We simulated a “Janus” micropost with four patches of alternating homeotropic and planar degenerate anchoring (Figure 4). Just like with the spherical Janus particle, we observe strong preference of the satellite particles to bind to the homeotropic patches on the post’s surface, and because the post cannot tilt, we obtain a stable state. The particle binds strongly to the middle of the homeotropic patch with a partial Saturn-ring arc, tethered to the seams between homeotropic and planar patches, while its free energy is the highest in the vicinity of the planar denegerate patch. The free energy profile (Figure 4b) reveals a torque that forces the particle into the stable configuration. Both the seams between the planar and homeotropic anchoring and the corner between the post and the cell walls are a high free-energy regions that traps defects. We observe a hysteresis effect when rotating the particle around the post: the tethered disclination line latches on to the lower or upper corner, creating a high-energy metastable state that can decay via thermal fluctuations or minute vertical movement of the particle, which we ignored in our simulations (Figure 4a).

## 3. Perspectives for Future Studies

Because the shape and stability of the disclinations are topologically protected, the choice of particle size, confinement, liquid crystal elastic anisotropy and strength of surface anchoring has little effect on the qualitative behavior of the Janus nematic coolids, but does affect the free energy landscape and thus interaction strength and kinetics of assembly. We explored this extensive phase (material) space only in a very narrow region; therefore, one could improve and fine-tune the desired phenomena. The conformation in the stable state depends more strongly on the surface morphology, relative distribution and size of the Janus patches. Further studies could reveal other interesting and potentially useful states with diminished symmetry and possibly a randomized lattice. For example, using particle types with three caps could prevent formation of straight homogeneous chains by breaking the symmetry. Larger clusters of such particles could still form complex structures of lower symmetry even if highly symmetric valence states were unstable. Such frustration and symmetry breaking could potentially lead to glassy states or quasi-crystals, which were already observed with colloidal platelets [21]. The proposed mixing of Janus particles with different surface patterns opens a combinatorial variety of possibilities for particle arrangement.

Using a fixed Janus post in the homeotropic cell (far field director is along z direction) to trap and bind particles via horizontal disclination arcs which we propose here, could be used on one hand as a structural seed and on the other hand as a micro-anchor for the colloidal aggregation. The surface pattern can be engineered to set the valence that promotes desired lattice geometry. A fixed lattice could provide a long-range order, while the satellite particles are still movable to provide switching. Alternatively, free-floating cylinders, confined to prevent turning, could provide translational freedom to build lattices, but without instability to rotation around a horizontal axis, providing the best of both worlds.

## 4. Modeling and Theory Section

Numerical modeling based on minimization of the phenomenological Landau-de Gennes free energy was uses as the central approach for modeling the described Janus nematic colloids. This approach was in recent years demonstrated to give qualitative and quantitative results when compared to experiments and in details described in the reference [22]. The approach is based on the free energy functional written in terms of the nematic order parameter tensor (Q-tensor), approximated on a discrete finite difference mesh. The free energy is minimized by using a quasi-time-step relaxation scheme that mimics real orientational dynamics in the absence of material flow. For homeotropic surface patches, the Rapini-Papoular type uniform surface anchoring free energy is assumed, whereas for degenerate planar anchoring patches the surface energy formulated by Fournier and Galatola [23] is used. In order to model the Janus particles with various surface morphologies surface patterned particles, logical conditions are set to discriminate between planar and homeotropic surface regions. Despite their spherical shape, beads with patterned surfaces are anisotropic with the anisotropy arising from the patterning. Their orientation with respect to the undisturbed (far-field) director is in general characterized by two angles or by just one if the surface patterning is rotationally symmetric.

In the simulations, the following material parameters, *A* = −1.72 × 10^6^ J/m^3^, *B* = −2.12 × 10^6^ J/m^3^, *C* = 1.73 × 10^6^ J/m^3^, *L* = 40 pN, characteristic for a typical (5CB) nematic liquid crystal described in the single-elastic constant approximation were used in the free energy of the form described in [22]. The surface anchoring strengths on both homeotropic and planar degenerate parts characterized by *W* = 10^−2^ J/m^2^ are in the limit of strong anchoring. The chosen value has been used in simulations of liquid crystal colloids before, with excellent agreement with experiments [14]. The particle diameters are 1 µm in Figure 1, Figure 2 and Figure 3 and 0.9 µm in Figure 4. The choice of parameters ensure the regime of the disclination loops that are stable against a collapse into point defects and the strong anchoring that keeps the disclinations away from the surface. Variations of particle size, anisotropy of elastic constants, and anchoring strength have only a minor effect on the general behavior of defects and interactions of the Janus particles, as long as the parameters allow for the realization of the same regime.

## 5. Conclusions

To conclude, the generalized Janus nematic colloids could be used as a novel route towards particle functionalized structures in nematic liquid crystal colloids. By designing the morphology or surface patches with homeotropic or planar surface anchoring, the elastic distortion of the nematic orientation field and inter-particle interaction potentials can be varied and engineered to high-complexity and spatial anisotropy. As a possible platform for valence-determined assembly of colloidal structures, a colloidal superstructure is shown, where two non-Janus particles bind to the bivalent Janus colloidal particle, by pinning their (Saturn-ring) defect loops to the tethering spots generated at the contact lines between the surface patches at the Janus particle. The capacity to control the valence and stability of free-floating spherical particles is hindered by a multitude of topological states. We avoid this problem by suggesting binding of colloidal particles to a fixed Janus post in a nematic cell, which forms a micro-seed and micro-anchor for the colloidal particles in such hybrid confined structures.

The nematic profiles of multi-valent Janus colloids are shown to be dominated by the emergence of topological defects, which are effective combinations of surface boojums, possibly spread into lines of reduced order, and bulk segments of +1/2 or −1/2 defect line, which typically form defect arcs. The topological analysis also demonstrated the ambiguity of the topological charge as a topological invariant in Janus nematic colloids, as it can acquire multiple values for one given particle type. 

Finally, the basic motivation for this research originates in assembling novel functional micro- and sub-micro-materials with unusual and possibly unique properties that could be used in a range of fields, from complex optics, photonics, metamaterials, to plasmonics and biotechnology. 
